# Supplementary material for: Insertases Scramble Lipids: Molecular Simulations of MTCH2
Source: bioRxiv. 2023 Dec 2:2023.08.14.553169. Originally published 2023 Aug 14. Preprint. [Version 2] doi: 10.1101/2023.08.14.553169 (PMC10462046; doi:10.1101/2023.08.14.553169)
Supplement: 1 [file NIHPP2023.08.14.553169v2-supplement-1.pdf]

bioRxiv preprint doi: https://doi.org/10.1101/2023.08.14.553169; this version posted December 2, 2023. The copyright holder for this preprint (which was not certified by peer review) is the author/funder, who has granted bioRxiv a license to display the preprint in perpetuity. It is made available under aCC-BY 4.0 International license.

Supplemental Information

Insertases Scramble Lipids: Molecular Simulations of MTCH2

Ladislav Bartoš,[†,‡] Anant K. Menon,[§] and Robert Vácha[*,†,‡,¶]

[†] CEITEC – Central European Institute of Technology, Masaryk University, Kamenice 753/5, 625 00 Brno, Czech Republic

[‡] National Centre for Biomolecular Research, Faculty of Science, Masaryk University, Kamenice 753/5, 625 00 Brno, Czech Republic

[§] Department of Biochemistry, Weill Cornell Medical College, New York, NY 10065, USA

[¶] Department of Condensed Matter Physics, Faculty of Science, Masaryk University, Kotlářská 267/2, 611 37 Brno, Czech Republic

* E-mail: robert.vacha@muni.cz

bioRxiv preprint doi: https://doi.org/10.1101/2023.08.14.553169; this version posted December 2, 2023. The copyright holder for this preprint (which was not certified by peer review) is the author/funder, who has granted bioRxiv a license to display the preprint in perpetuity. It is made available under aCC-BY 4.0 International license.

Table S1: Distribution of umbrella sampling windows along the collective variable with biasing force constants used for Martini 3 simulations of lipid flip-flop through membrane with MTCH2. Reference distances are in nm, force constants in kJ mol^{-1} nm^{-2}.

Reference distance	Force constant	Reference distance	Force constant
2.00	100	−0.50	200
1.80	100	−0.60	200
1.60	100	−0.70	200
1.40	100	−0.80	200
1.30	200	−0.85	400
1.20	200	−0.90	400
1.10	200	−0.95	400
1.00	200	−1.00	400
0.90	200	−1.05	400
0.80	200	−1.10	400
0.70	200	−1.15	400
0.60	200	−1.20	400
0.50	200	−1.25	400
0.40	200	−1.30	400
0.30	200	−1.40	200
0.20	200	−1.50	200
0.10	200	−1.60	200
0.00	200	−1.70	200
−0.10	200	−1.80	200
−0.20	200	−1.90	200
−0.30	200	−2.00	100
−0.40	200	−2.10	100

bioRxiv preprint doi: https://doi.org/10.1101/2023.08.14.553169; this version posted December 2, 2023. The copyright holder for this preprint (which was not certified by peer review) is the author/funder, who has granted bioRxiv a license to display the preprint in perpetuity. It is made available under aCC-BY 4.0 International license.

Table S2: Distribution of umbrella sampling windows along the collective variable with biasing force constants used for Martini 2 simulations of lipid flip-flip through membrane with MTCH2. Reference distances are in nm, force constants in kJ mol^{-1} nm^{-2}.

Reference distance	Force constant	Reference distance	Force constant
2.10	500	−0.70	1000
2.00	500	−0.75	1000
1.90	500	−0.80	1000
1.80	500	−0.85	2000
1.70	500	−0.90	2000
1.60	500	−0.95	2000
1.50	500	−1.00	2000
1.40	500	−1.04	3000
1.30	500	−1.06	3000
1.20	500	−1.08	3000
1.10	500	−1.10	3000
1.00	500	−1.12	3000
0.90	500	−1.14	3000
0.80	500	−1.16	3000
0.70	500	−1.18	3000
0.60	500	−1.20	3000
0.50	500	−1.22	3000
0.40	500	−1.25	2000
0.30	500	−1.30	2000
0.20	500	−1.35	2000
0.10	500	−1.40	2000
0.00	500	−1.45	1000
−0.10	500	−1.50	1000
−0.20	500	−1.55	1000
−0.30	500	−1.60	1000
−0.35	1000	−1.65	1000
−0.40	1000	−1.70	500
−0.45	1000	−1.80	500
−0.50	1000	−1.90	500
−0.55	1000	−2.00	500
−0.60	1000	−2.10	500
−0.65	1000	−2.20	500

bioRxiv preprint doi: https://doi.org/10.1101/2023.08.14.553169; this version posted December 2, 2023. The copyright holder for this preprint (which was not certified by peer review) is the author/funder, who has granted bioRxiv a license to display the preprint in perpetuity. It is made available under aCC-BY 4.0 International license.

Table S3: Distribution of umbrella sampling windows along the collective variable with biasing force constants used for Martini 2 and 3 simulations of lipid flip-flop through proteinless membrane. Reference distances are in nm, force constants in kJ mol^{-1} nm^{-2}.

Reference distance	Force constant	Reference distance	Force constant
2.30	1000	−0.05	4000
2.20	1000	−0.10	4000
2.10	1000	−0.15	3000
2.00	1000	−0.20	3000
1.90	1000	−0.25	3000
1.80	1000	−0.30	3000
1.70	1000	−0.35	2000
1.60	1000	−0.40	2000
1.50	1000	−0.45	2000
1.40	1000	−0.50	2000
1.30	1000	−0.55	2000
1.20	1000	−0.60	2000
1.10	1000	−0.65	2000
1.00	2000	−0.70	2000
0.95	2000	−0.75	2000
0.90	2000	−0.80	2000
0.85	2000	−0.85	2000
0.80	2000	−0.90	2000
0.75	2000	−0.95	2000
0.70	2000	−1.00	2000
0.65	2000	−1.10	1000
0.60	2000	−1.20	1000
0.55	2000	−1.30	1000
0.50	2000	−1.40	1000
0.45	2000	−1.50	1000
0.40	2000	−1.60	1000
0.35	2000	−1.70	1000
0.30	2000	−1.80	1000
0.25	3000	−1.90	1000
0.20	3000	−2.00	1000
0.15	3000	−2.10	1000
0.10	4000	−2.20	1000
0.05	4000	−2.30	1000
0.00	4000		

bioRxiv preprint doi: https://doi.org/10.1101/2023.08.14.553169; this version posted December 2, 2023. The copyright holder for this preprint (which was not certified by peer review) is the author/funder, who has granted bioRxiv a license to display the preprint in perpetuity. It is made available under aCC-BY 4.0 International license.

Table S4: Position and dihedral restraints applied during the individual stages of atomistic equilibration. All values are in kJ mol^{-1} nm^{-2} or kJ mol^{-1} rad^{-2} (for lipid dihedrals).

	protein backbone	protein sidechains	lipid phosphorus	lipid dihedrals
I	4000	2000	1000	1000
II	2000	1000	400	400
III	1000	500	400	200
IV	500	200	200	200
V	200	50	40	100
VI	50	0	0	0

bioRxiv preprint doi: https://doi.org/10.1101/2023.08.14.553169; this version posted December 2, 2023. The copyright holder for this preprint (which was not certified by peer review) is the author/funder, who has granted bioRxiv a license to display the preprint in perpetuity. It is made available under aCC-BY 4.0 International license.

Table S5: Distribution of umbrella sampling windows along the collective variable with biasing force constants used for atomistic simulations of lipid flip-flop through proteinless membrane. ^{R} marks windows where Hamiltonian replica exchange was applied. Reference distances are in nm, force constants in kJ mol^{-1} nm^{-2}.

Reference distance	Force constant	Reference distance	Force constant
2.30	1000	−0.03	2000^R
2.20	1000	−0.06	2000^R
2.10	1000	−0.09	2000^R
2.00	1000	−0.12	2000^R
1.90	1000	−0.15	2000^R
1.80	1000	−0.18	2000^R
1.70	1000	−0.21	2000^R
1.60	1000	−0.25	2000^R
1.50	1000	−0.30	1000
1.40	1000	−0.40	1000
1.30	1000	−0.50	1000
1.20	1000	−0.60	1000
1.10	1000	−0.70	1000
1.00	1000	−0.80	1000
0.90	1000	−0.90	1000
0.80	1000	−1.00	1000
0.70	1000	−1.10	1000
0.60	1000	−1.20	1000
0.50	1000	−1.30	1000
0.40	1000	−1.40	1000
0.30	1000	−1.50	1000
0.25	2000	−1.60	1000
0.21	2000^R	−1.70	1000
0.18	2000^R	−1.80	1000
0.15	2000^R	−1.90	1000
0.12	2000^R	−2.00	1000
0.09	2000^R	−2.10	1000
0.06	2000^R	−2.20	1000
0.03	2000^R	−2.30	1000
0.00	2000^R		

bioRxiv preprint doi: https://doi.org/10.1101/2023.08.14.553169; this version posted December 2, 2023. The copyright holder for this preprint (which was not certified by peer review) is the author/funder, who has granted bioRxiv a license to display the preprint in perpetuity. It is made available under aCC-BY 4.0 International license.

Table S6: Distribution of umbrella sampling windows along the collective variable used for atomistic simulations of lipid flip-flop through membrane with MTCH2. "Origin" corresponds to the pulling simulation from which the initial configuration for the given umbrella sampling window was taken. ^{R} marks windows where Hamiltonian replica exchange was applied. Reference distances are in nm, force constants in kJ mol^{-1} nm^{-2}.

Reference distance	Force constant	Origin	Reference distance	Force constant	Origin
2.20	1000	pull 1	−0.90	1000	pull 2^R
2.10	1000	pull 1	−0.92	3000	pull 3^R
2.00	1000	pull 1	−0.94	3000	pull 2^R
1.90	1000	pull 1	−0.96	3000	pull 3^R
1.80	1000	pull 1	−0.98	3000	pull 2^R
1.70	1000	pull 1	−1.00	3000	pull 3^R
1.60	1000	pull 1	−1.02	3000	pull 2^R
1.50	1000	pull 1	−1.04	3000	pull 3^R
1.40	1000	pull 1	−1.06	3000	pull 2^R
1.30	1000	pull 1	−1.08	3000	pull 3^R
1.20	1000	pull 1	−1.10	3000	pull 2^R
1.10	1000	pull 1	−1.12	3000	pull 3^R
1.00	1000	pull 1	−1.14	3000	pull 2^R
0.90	1000	pull 1	−1.16	3000	pull 3^R
0.80	1000	pull 1	−1.18	3000	pull 2^R
0.70	1000	pull 1	−1.20	3000	pull 3^R
0.60	1000	pull 1	−1.22	3000	pull 2^R
0.50	1000	pull 1	−1.24	3000	pull 3^R
0.40	1000	pull 1	−1.26	3000	pull 2^R
0.30	1000	pull 1	−1.28	3000	pull 3^R
0.20	1000	pull 1	−1.30	3000	pull 2^R
0.10	1000	pull 1	−1.32	3000	pull 3^R
0.00	1000	pull 1	−1.34	3000	pull 2^R
−0.05	1000	pull 1	−1.36	3000	pull 3^R
−0.10	1000	pull 1	−1.38	3000	pull 2^R
−0.15	1000	pull 1	−1.40	1000	pull 3^R
−0.20	1000	pull 1	−1.45	1000	pull 2^R
−0.25	1000	pull 1	−1.50	1000	pull 3^R
−0.30	1000	pull 1	−1.55	1000	pull 2^R
−0.35	1000	pull 1	−1.60	1000	pull 3^R
−0.40	1000	pull 1	−1.65	1000	pull 2^R
−0.45	1000	pull 1	−1.70	1000	pull 3^R
−0.50	1000	pull 2^R	−1.75	1000	pull 2^R
−0.55	1000	pull 3^R	−1.80	1000	pull 3^R
−0.60	1000	pull 2^R	−1.85	1000	pull 2^R
−0.65	1000	pull 3^R	−1.90	1000	pull 3^R
−0.70	1000	pull 2^R	−1.95	1000	pull 2^R
−0.75	1000	pull 3^R	−2.00	500	pull 3^R
−0.80	1000	pull 2^R	−2.10	500	pull 2^R
−0.85	1000	pull 3^R	−2.20	500	pull 3^R

bioRxiv preprint doi: https://doi.org/10.1101/2023.08.14.553169; this version posted December 2, 2023. The copyright holder for this preprint (which was not certified by peer review) is the author/funder, who has granted bioRxiv a license to display the preprint in perpetuity. It is made available under aCC-BY 4.0 International license.

A

B

C

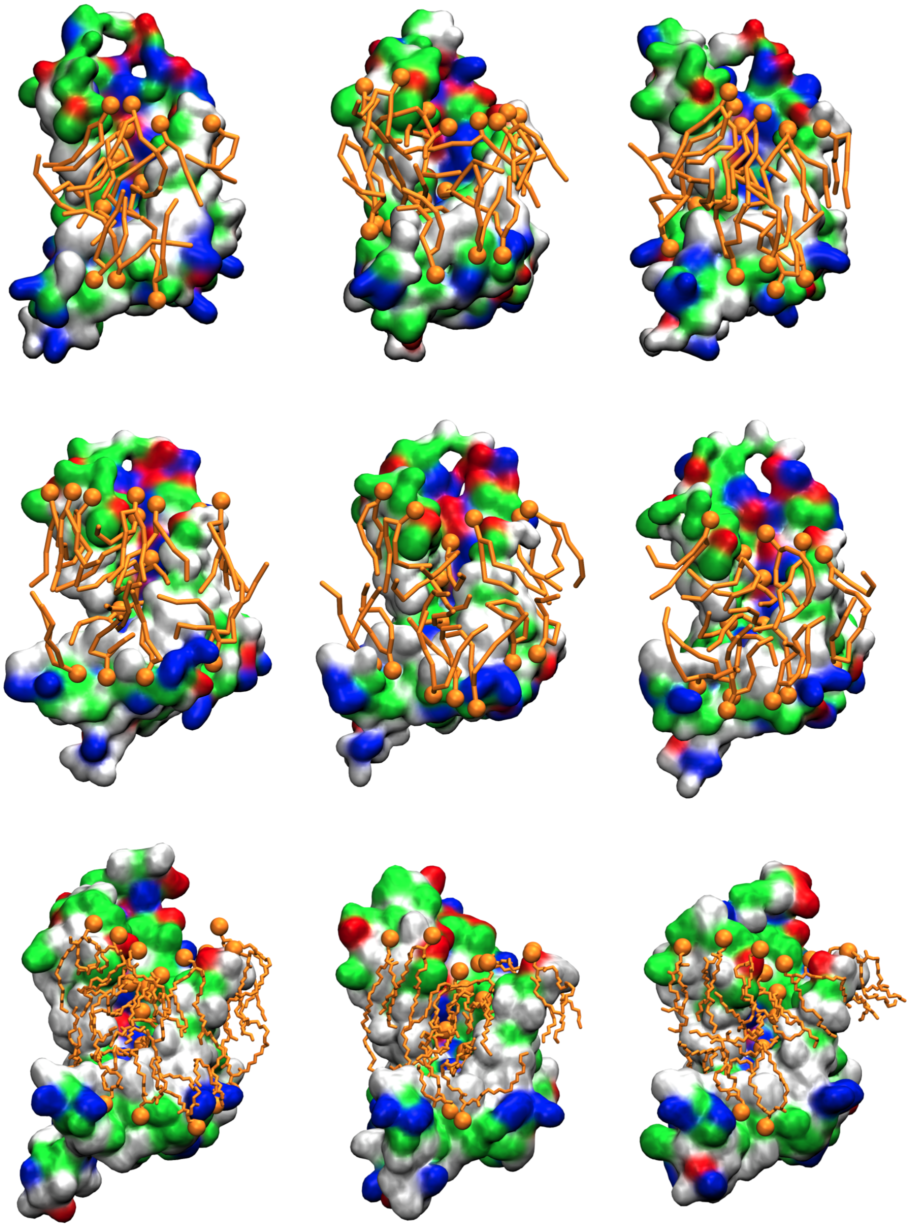

Figure S1: Simulation snapshots obtained from unbiased Martini 3 (A), Martini 2 (B), and atomistic (C) simulations showing insertion of lipid headgroups into the hydrophilic cavity of MTCH2 and the disorder in the lipid tails. Only lipids in proximity to the cavity are shown. Lipid headgroups are depicted as orange beads while lipid tails as orange tubes. MTCH2 is represented as a molecular surface colored according to the character of its residues (hydrophilic = green, hydrophobic = white, positively charged = blue, negatively charged = red).

bioRxiv preprint doi: https://doi.org/10.1101/2023.08.14.553169; this version posted December 2, 2023. The copyright holder for this preprint (which was not certified by peer review) is the author/funder, who has granted bioRxiv a license to display the preprint in perpetuity. It is made available under aCC-BY 4.0 International license.

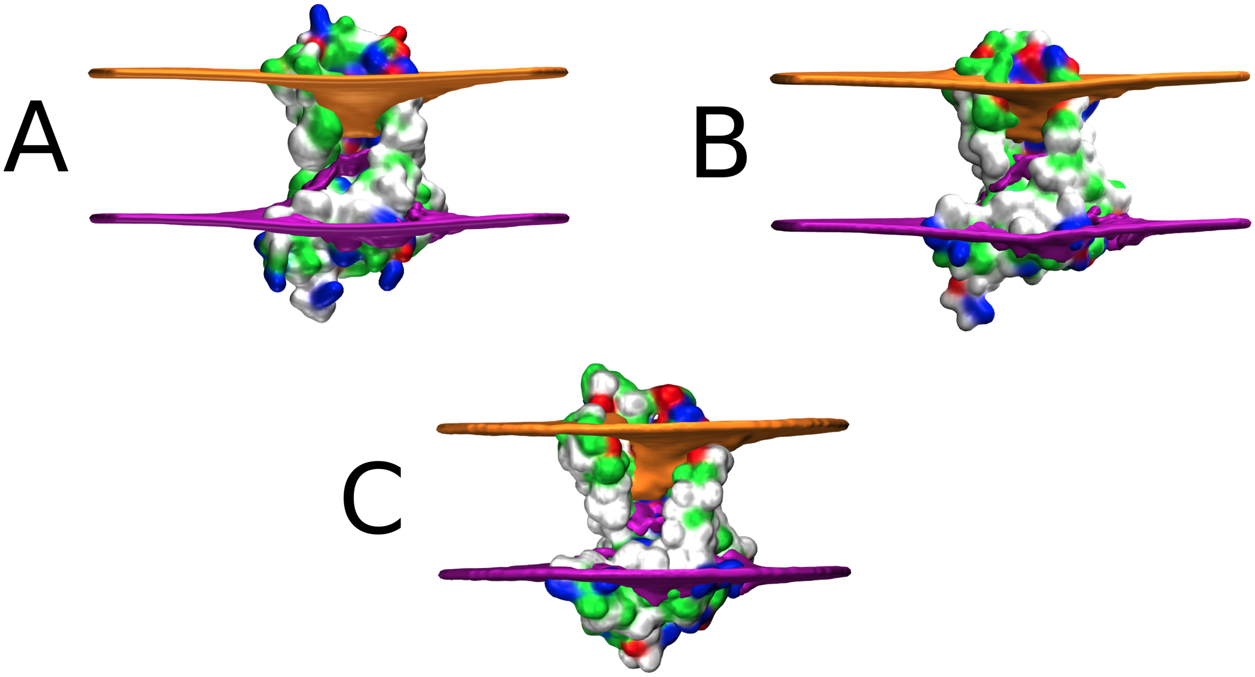

Figure S2: Membrane thickness in the vicinity of MTCH2 calculated from unbiased Martini 3 (A), Martini 2 (B), and atomistic (C) simulations. The orange and purple surfaces represent the average positions of lipid phosphate beads (or phosphorus atoms, in case of atomistic simulation) in the upper and lower leaflet, respectively. The membrane is dramatically thinned in the cavity of MTCH2 in all three force fields.

bioRxiv preprint doi: https://doi.org/10.1101/2023.08.14.553169; this version posted December 2, 2023. The copyright holder for this preprint (which was not certified by peer review) is the author/funder, who has granted bioRxiv a license to display the preprint in perpetuity. It is made available under aCC-BY 4.0 International license.

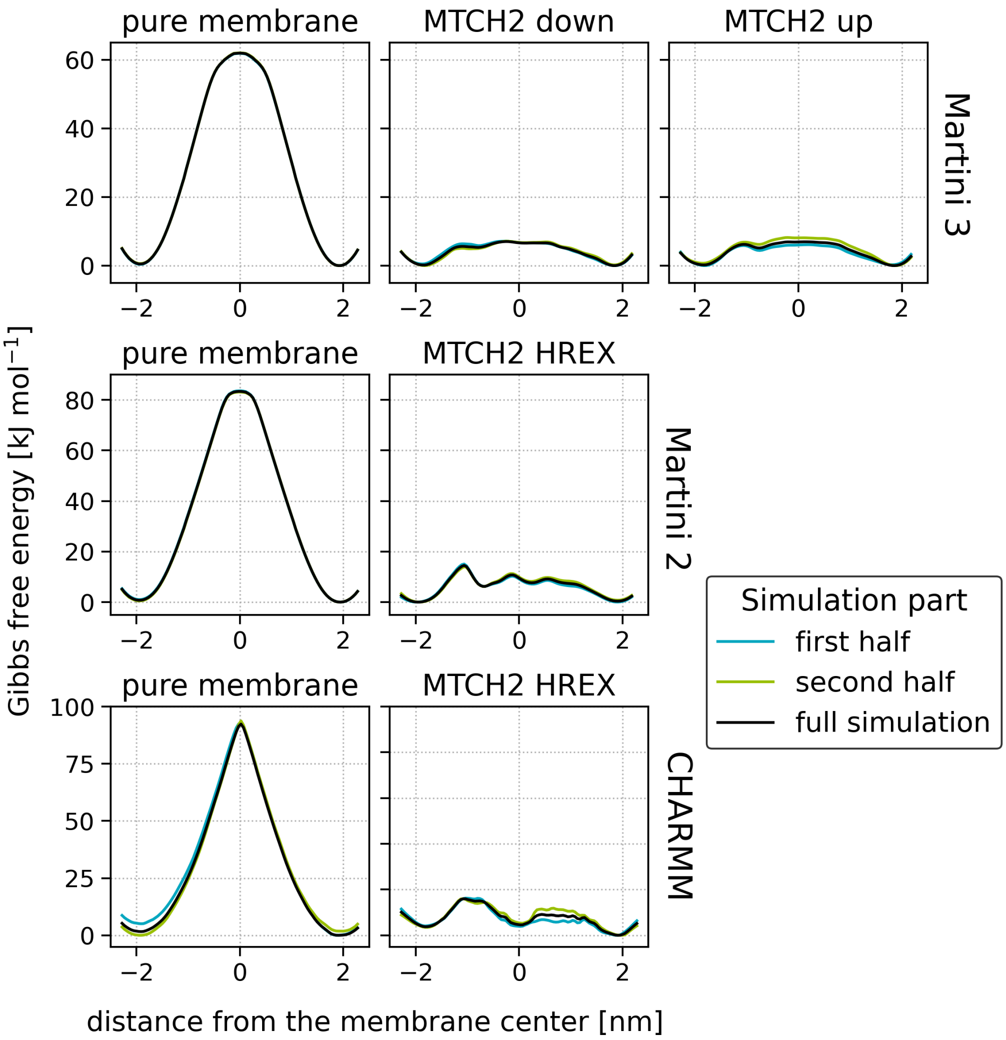

Figure S3: Convergence of free energy calculations captured by calculating additional free energy profiles from the first (azure) and the second (lime) halves of the production phase of umbrella sampling. "MTCH2 down" and "MTCH2 up" correspond to umbrella sampling simulations in which MTCH2 was present, and the initial configurations for the umbrella sampling windows were obtained from steered molecular dynamics, during which the selected lipid phosphate was pulled from the upper to the lower leaflet and from the lower to the upper leaflet, respectively. "MTCH2 HREX" corresponds to simulation in which umbrella sampling was enhanced by Hamiltonian replica exchange. For more details, refer to the Methods section. Note that an additional estimate of the error can be obtained from the asymmetry of the free energy minima located on each side of the profile.

bioRxiv preprint doi: https://doi.org/10.1101/2023.08.14.553169; this version posted December 2, 2023. The copyright holder for this preprint (which was not certified by peer review) is the author/funder, who has granted bioRxiv a license to display the preprint in perpetuity. It is made available under aCC-BY 4.0 International license.

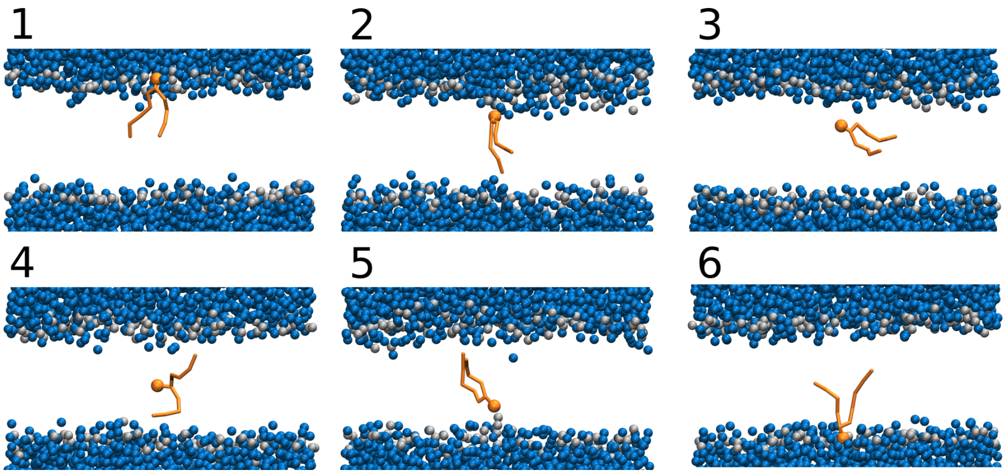

Figure S4: Representative simulation snapshots of a selected lipid translocating through a proteinless membrane captured from Martini 3 umbrella sampling windows. The snapshots are centered on the phosphate of the translocating lipid. The translocating lipid is shown in orange, while only phosphate groups are shown for the other lipids, represented by gray beads. Only water beads close to the membrane are shown, and these are depicted as blue beads.

bioRxiv preprint doi: https://doi.org/10.1101/2023.08.14.553169; this version posted December 2, 2023. The copyright holder for this preprint (which was not certified by peer review) is the author/funder, who has granted bioRxiv a license to display the preprint in perpetuity. It is made available under aCC-BY 4.0 International license.

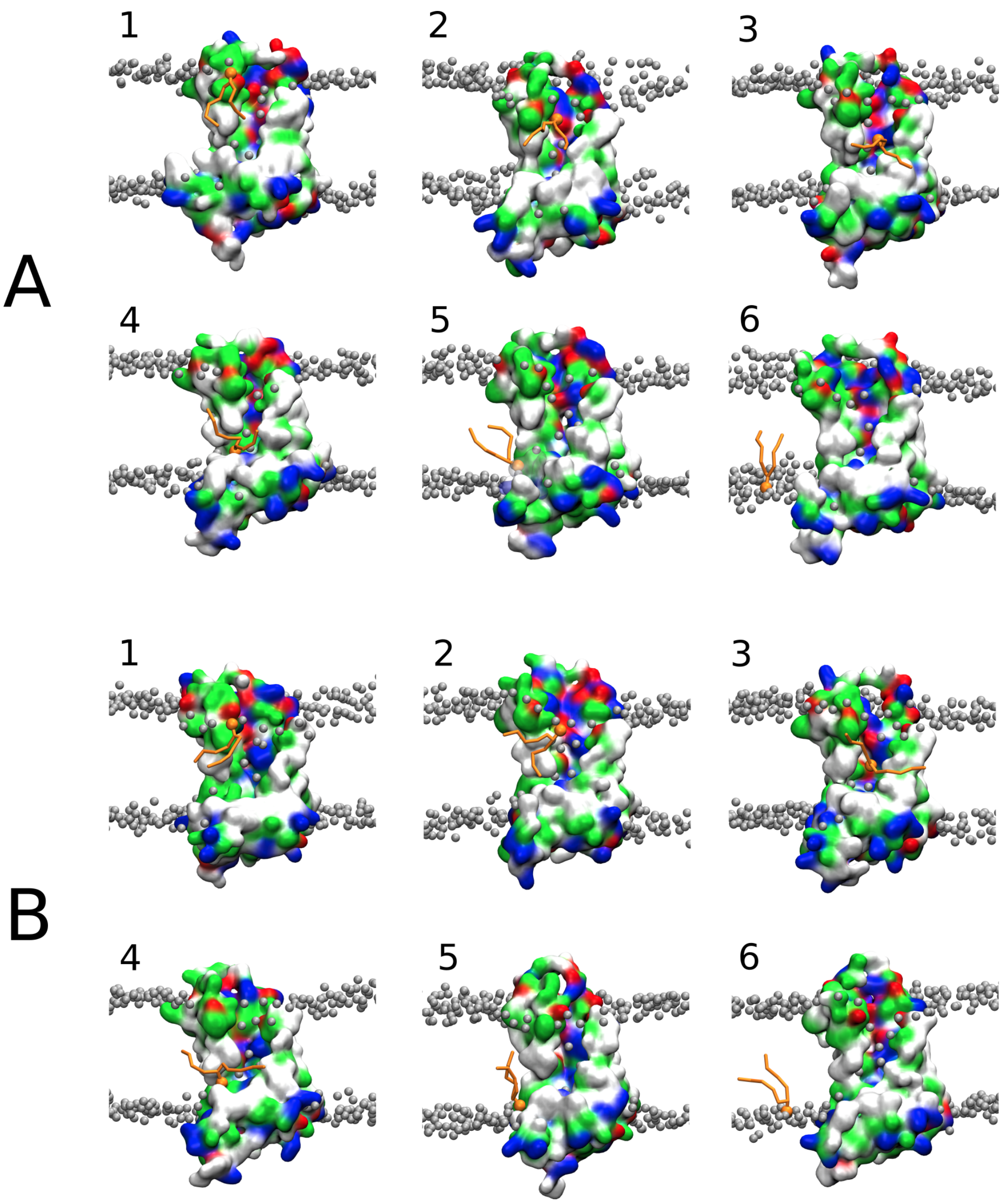

Figure S5: Representative simulation snapshots captured from Martini 3 umbrella sampling windows. The initial configurations for the umbrella sampling windows were obtained from steered molecular dynamics (MD) simulations which involved the pulling of a selected lipid phosphate either from the upper leaflet to the lower leaflet (A) or from the lower leaflet to the upper leaflet (B). MTCH2 is depicted as a molecular surface colored according to the character of its residues (hydrophilic = green, hydrophobic = white, positively charged = blue, negatively charged = red). The pulled lipid is shown in orange, while other lipids are only partially represented with their phosphate groups illustrated as gray beads. Water and ions are not shown.

bioRxiv preprint doi: https://doi.org/10.1101/2023.08.14.553169; this version posted December 2, 2023. The copyright holder for this preprint (which was not certified by peer review) is the author/funder, who has granted bioRxiv a license to display the preprint in perpetuity. It is made available under aCC-BY 4.0 International license.

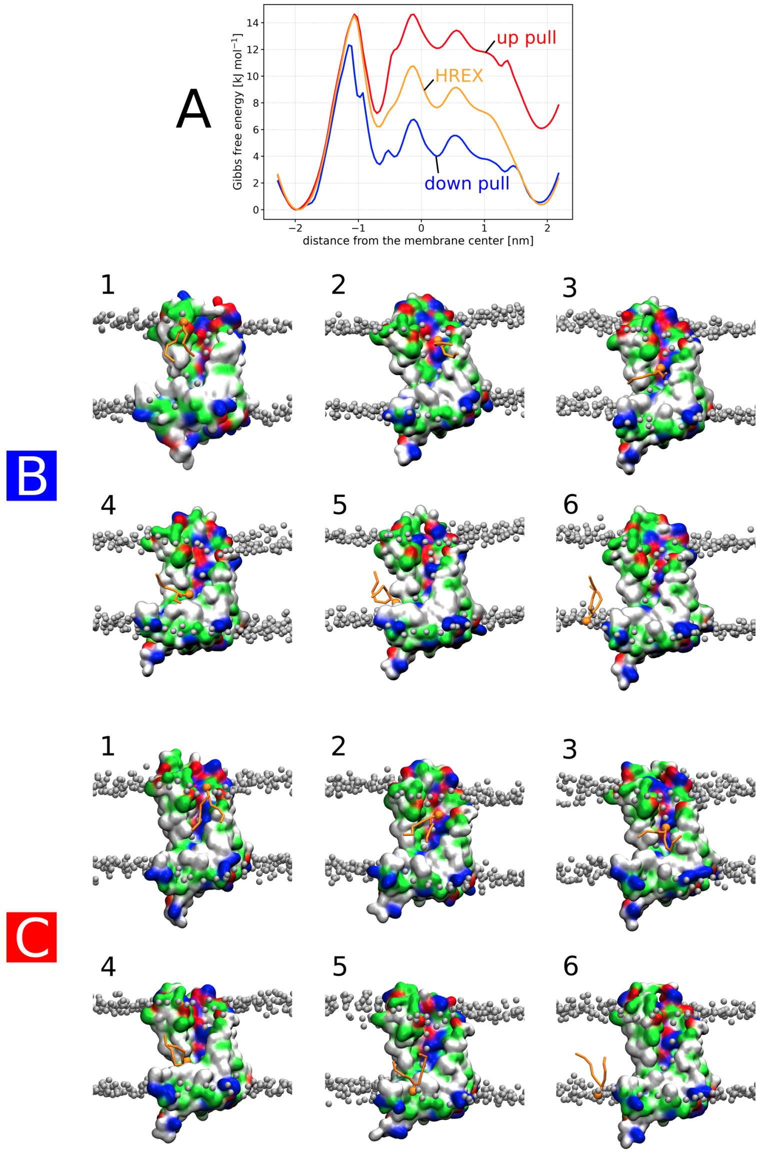

Figure S6: A) Comparison of free energy profiles calculated using umbrella sampling windows with initial configurations obtained from steered molecular dynamics simulations, where the selected lipid phosphate was either pulled from the upper to the lower leaflet (down pull, blue profile) or from the lower to the upper leaflet (up pull, red profile). The HREX, orange profile was calculated using umbrella sampling windows from both pulling directions (by alternating the origin of initial configurations) while employing Hamiltonian replica exchange. Note the hysteresis between the red and blue profiles, which prompted the use of Hamiltonian replica exchange. B–C) Representative simulation snapshots captured from Martini 2 umbrella sampling windows. B corresponds to the set of windows originating from the "down pull", while C corresponds to windows originating from the "up pull". "HREX" windows are not shown. The color scheme is the same as in Figure S5. The difference between the "down pull" and "up pull" profiles likely originates from the lipid using a different scrambling pathway in the C-terminal part of the protein when pulled from the lower to the upper leaflet (compare snapshots B5 and C5). Note that this alternative pathway is likely an artifact of pulling, as it was not observed in the windows with applied Hamiltonian replica exchange after initial equilibration.

bioRxiv preprint doi: https://doi.org/10.1101/2023.08.14.553169; this version posted December 2, 2023. The copyright holder for this preprint (which was not certified by peer review) is the author/funder, who has granted bioRxiv a license to display the preprint in perpetuity. It is made available under aCC-BY 4.0 International license.

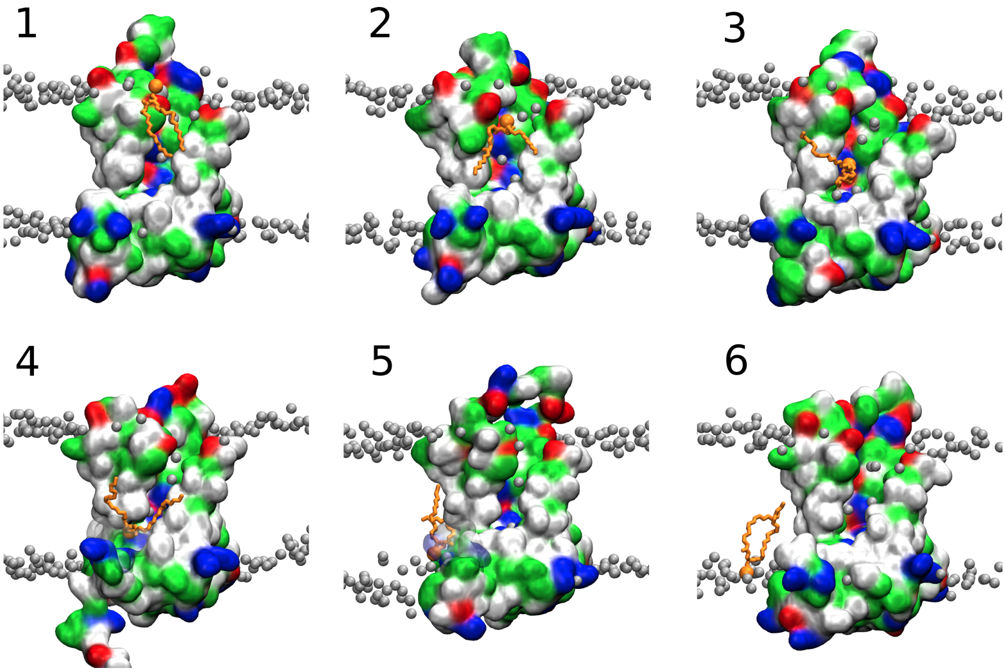

Figure S7: Representative simulation snapshots of a selected lipid translocating through a membrane containing MTCH2 captured from atomistic umbrella sampling windows. MTCH2 is depicted as a molecular surface colored according to the character of its residues (hydrophilic = green, hydrophobic = white, positively charged = blue, negatively charged = red). The pulled lipid is shown in orange, while other lipids are only partially represented with their phosphate groups illustrated as gray beads. Water and ions are not shown.
